# Research on Performance Enhancement, Output Regulation, and the Applications of Nanogenerators

**DOI:** 10.3390/mi16020208

**Published:** 2025-02-12

**Authors:** You-Jun Huang, Chen-Kuei Chung

**Affiliations:** Department of Mechanical Engineering, National Cheng Kung University, Tainan 701, Taiwan

The demand for sensors in wearable devices [1,2] and the Internet of Things (IoT) [3,4] is increasing rapidly with the advancement of technology. Traditional mechanical sensors encounter limitations in battery life, materials, and structure, making it difficult for them to meet the requirements of large-area, long-term sensing, as well as high flexibility and high stretchability in strict conditions. Therefore, finding alternative sensing solutions has become crucial. Nanogenerators [5,6] are devices that convert mechanical energy into electrical energy. Triboelectric nanogenerators (TENGs) [7,8] and piezoelectric nanogenerators (PENGs) [9,10] are common nanogenerators. The characteristics of their output electrical signals change with the force applied, as well their generation of electrical energy, making them suitable for development as self-powered mechanical sensors. Therefore, TENG and PENG are attractive for applications to human motion sensing [11,12] and IoT. They provide solutions to the limitations of traditional mechanical sensors. TENG works by exploiting differences in the ability of two materials to capture and release charges. When the materials undergo contact, separation, or parallel motion, charge transfers and induced charges occur. When the back sides of the materials are connected to an external circuit, current flows through the circuit to maintain the electrical neutrality of the materials [7]. On the other hand, PENG works by utilizing electrostatic dipoles in the materials, which changes their dipole moment when subjected to pressure and subsequently released. This change creates a potential difference between the two sides of the material, generating an electrical current [9].

Currently, the primary methods for enhancing the electrical output performance of TENGs focus on increasing the effective contact area [13] of the materials [14] and altering their dielectric and conductive properties. In the past, to achieve a better electrical output performance or mechanical sensitivity, scientists often used high-cost fabrication techniques and expensive or difficult-to-obtain materials. However, with the addition of low-cost, high-speed, and large-area fabrication methods, as well as more accessible materials and diverse material modification techniques, the cost of TENG- and PENG-based mechanical sensors has gradually decreased, greatly improving their feasibility for widespread adoption [15,16]. By combining multiple identical nanogenerators, the electrical output performance can be enhanced, increasing the generated electrical energy and the versatility of the sensors. Moreover, combining different types of nanogenerators not only improves the accuracy of the sensing, but also resolves the sensing blind spots of individual devices, making them applicable in various scenarios. With the assistance of external circuits, the noise in the electrical signals generated by nanogenerators can be further filtered out, leaving only the most important information [17]. This makes it easier for computers or microcontroller units (MCUs) to process and utilize the data. Nowadays, mechanical sensors made from TENGs and PENGs have already been proven to be applicable in areas such as human–machine interface sensing [18], acoustic sensing, fluid sensing [19], and micro-wearable sensing [20], as well as in small electronic devices.

This Special Issue includes a number of articles, each contributing to the advancement of nanogenerator research and its potentially practical applications. In pursuit of enhancing the output performance of nanogenerators, several studies made significant progress. For example, Abdel-Rahman et al. [21] developed nano-groove and prism-structured TENGs by employing cost-efficient laser processing and molding techniques, providing a scalable approach to improving device efficiency. Through the design of specialized mechanisms, it may effectively overcome several limitations of nanogenerators, allowing them to capture a wider range of diverse and dynamic kinetic signals. Sepúlveda et al. [22] designed a flexible and biocompatible nanogenerator capable for detecting motion signals from biological bending and releasing motions, showcasing its potential for medical and wearable applications. Ahn et al. [23] presented a single-electrode flowing liquid-based TENG to measure fluid flow rate and velocity with high precision. Signal processing and output circuit enhancements have also played a critical role in advancing TENG applications. Deng et al. [24] introduced an operator-based voltage control method to refine TENG outputs, making the signals more interpretable and suitable for practical uses. Yang et al. [25] successfully combined TENGs with a miniaturized power supply (MPS), enabling autonomous operation and expanding their usability across various fields. Collectively, these studies mark significant progress in enhancing the versatility, efficiency, and practical applications of nanogenerators, particularly in wearable devices, IoT systems, and broader human-centered technologies. In addition to the studies mentioned above, the *Micromachines* journal has also compiled a diverse range of research on nanogenerators applied across various fields. The potential of these studies to impact daily life and industrial processes addresses the growing relevance of nanogenerator activity in addressing real-world challenges.
